# The snake-head sign on MRI in craniocervical junction dural arteriovenous fistulas: a novel imaging feature and a hypothesis of venous remodeling and congestion

**DOI:** 10.3389/fneur.2026.1972107

**Published:** 2026-10-16

**Authors:** Dongchao Shen, Xinghua Chen, Zhe Zhang, Yanying Wang, Chang Geng, Hongzhi Guan

**Affiliations:** 1 Department of Neurology, Peking Union Medical College Hospital, Chinese Academy of Medical Sciences and Peking Union Medical College, Beijing, China; 2 Department of Neurology, Linyi Shangcheng Hospital, Linyi, China

**Keywords:** craniocervical junction, dural arteriovenous fistula, MRI, snake-head sign, venous hypertensive myelopathy

## Abstract

**Background:**

Craniocervical junction (CCJ)-region dural arteriovenous fistulas (dAVFs) are rare but potentially reversible causes of venous congestive myelopathy, often difficult to recognize due to non-specific presentation. We observed a characteristic pattern of intramedullary T2 hyperintensity on sagittal MRI, involving the medulla and upper cervical cord with a rostral end divided into ventral and dorsal limbs, which we termed the “snake-head sign.” This study aimed to characterize the prevalence of this feature among reported CCJ-region dAVF cases and explore its clinical and anatomical correlates.

**Methods:**

We retrospectively reviewed published cases of CCJ-region or adjacent dAVFs reported between 1996 and 2026 that included retrievable sagittal T2-weighted or FLAIR images. The presence of the snake-head sign was assessed, and clinical, angiographic, and treatment-related variables were extracted, including fistula location, feeding artery, draining vein direction, treatment modality, and outcome. Associations with the sign were evaluated using univariate analyses. Given the retrospective, literature-based design and limited sample size, findings were considered hypothesis-generating.

**Results:**

Among 64 reported cases, the snake-head sign was identified in 31 (48.4%). No significant associations were found between the sign and age, sex, symptom duration, feeding artery, treatment modality, or outcome. However, a non-significant trend was observed for venous drainage configuration: cases with cranial convergence of the draining vein toward the CCJ more frequently demonstrated the sign, whereas purely caudal drainage patterns showed it less often. Fistulas at lower cervical nerve-root sleeves, requiring a longer cranial venous course, also showed higher sign frequency. These findings suggest the sign may reflect venous outflow geometry rather than arterial source or fistula location.

**Conclusion:**

The snake-head sign is a recognizable MRI feature present in roughly half of reported CCJ-region dAVF cases. Its identification may raise clinical suspicion for CCJ-related dAVF, particularly in unexplained venous congestive myelopathy. The trend toward a higher frequency of the sign in cases with cranially convergent venous drainage raises the hypothesis that it reflects concentrated venous congestion at this anatomical transition zone. Prospective multicenter studies with standardized imaging review and angiographic correlation are needed to confirm its diagnostic value and clarify the underlying mechanism.

## Introduction

Dural arteriovenous fistulas (dAVFs) involving the craniocervical junction (CCJ) are uncommon but potentially reversible causes of venous congestive myelopathy (1, 2). Because clinical manifestations are often non-specific and may overlap with inflammatory, degenerative, or other non-vascular myelopathies, diagnosis is frequently delayed (2, 3). Although MRI may reveal characteristic abnormalities such as spinal cord edema and abnormal perimedullary vessels, specific imaging features that facilitate early recognition of CCJ-region dAVFs remain insufficiently characterized (4, 5).

In our clinical practice, we observed a distinctive pattern of intramedullary signal abnormality on sagittal MRI in a subset of patients with CCJ-related dAVFs. This finding consists of an intramedullary T2 hyperintensity extending from the upper cervical cord into the medulla, whose rostral end is divided into ventral and dorsal limbs separated by relatively spared central parenchyma, resembling the open mouth of a snake. We refer to it as the “snake-head sign” (outlined in red in Figure 1). This configuration appears to represent a visually recognizable pattern of venous congestive edema at the CCJ. However, its prevalence, clinical context, and potential anatomical basis have not, to our knowledge, been systematically characterized.

**Figure 1:**
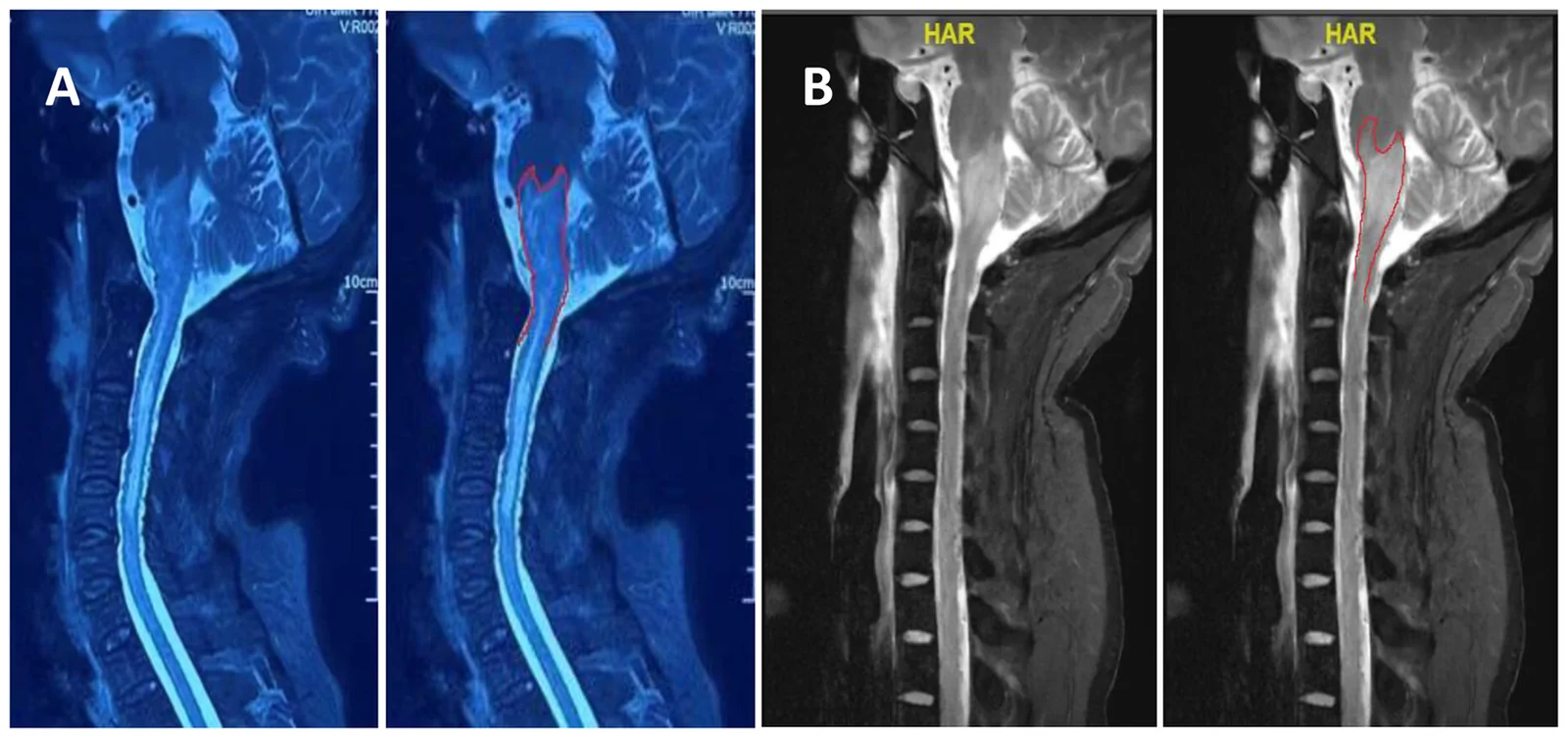
Two Cases of Dural Arteriovenous Fistula Presenting with a Typical “Snake-Head Sign” in our center. **(A)** Male, 46 years old, presenting with a chief complaint of numbness in the extremities for 10 days, diagnosed with a dural arteriovenous fistula at the petrous apex. **(B)** Female, 40 years old, presenting with a chief complaint of intermittent dizziness and vomiting for 23 days, with worsening symptoms and dyspnea for 3 days, diagnosed with a left posterior fossa dural arteriovenous fistula.

The objective of this study was to characterize the occurrence of the snake-head sign among published cases of CCJ-region and adjacent dAVFs, to describe the clinical and angiographic context in which this imaging feature appears, and to explore a potential anatomical and hemodynamic mechanism underlying its formation. Given the rarity of this condition and the limitations of available case-based data, this study was designed as a hypothesis-generating analysis rather than a confirmatory investigation.

## Methods

### Case source and assessment of the snake-head sign

A retrospective literature-based analysis was performed to identify published individual cases of dAVF with involvement of the CCJ venous system. The search strategy combined the following terms: (“dural arteriovenous fistula” OR “radicular arteriovenous fistula” OR “spinal dural arteriovenous fistula”) AND (“craniovertebral junction” OR “craniocervical junction” OR “occipitocervical junction”), in order to maximize search sensitivity while maintaining relevance to the anatomic region of interest. The search was limited to articles published between January 1, 1990, and May 31, 2026, and restricted to those written in English. Reference lists of included studies were manually reviewed to identify additional eligible cases.

Cases were included when sagittal T2-weighted or FLAIR images covering the medulla and upper cervical cord were available and of sufficient quality to determine the presence or absence of the snake-head sign. Cases illustrated only by angiography, axial images, or contrast-enhanced sequences were excluded. Rather than restricting inclusion solely according to the anatomical site of the fistulous point, we included cases in which abnormal venous drainage involved the CCJ region, including fistulas located at the CCJ, adjacent intracranial dural sinuses, or lower cervical nerve-root sleeves. Cases without evaluable sagittal imaging, isolated intracranial lesions without CCJ venous involvement, and hemorrhagic vascular lesions were excluded. Finally, 54 articles published between 1996 and 2026 were included (Figure 2 and Supplementary material). For each case the following were recorded: source/year, age, sex, chief complaint, symptom duration, fistula location, feeding artery, draining vein, MRI findings, treatment, and clinical outcome.

**Figure 2:**
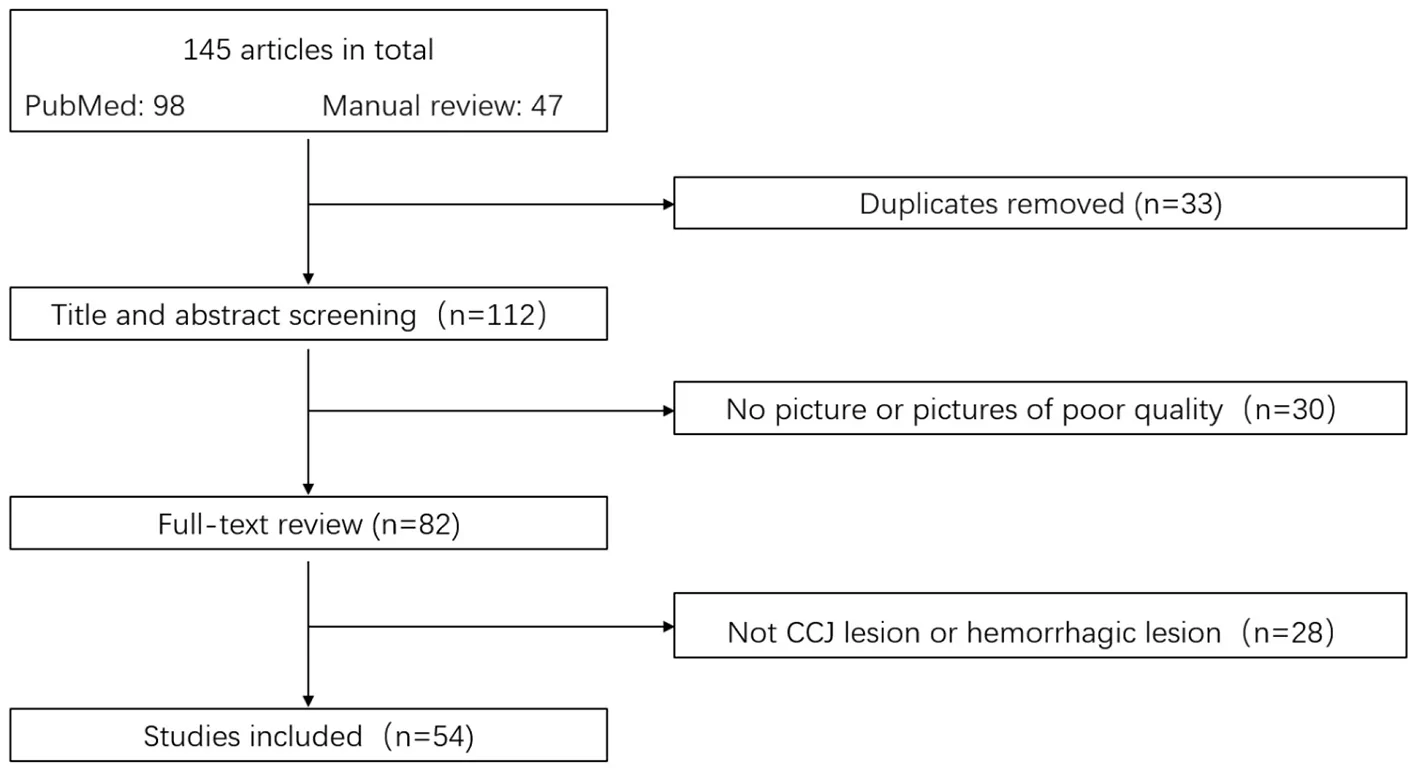
The flow chart of the literature search in this article.

Two investigators (DCS and XHC) reviewed the published sagittal T2-weighted or FLAIR images of each case for the presence of the snake-head sign, defined as a longitudinally extensive intramedullary hyperintensity involving the upper cervical cord and extending rostrally into the medulla, whose rostral end is divided into ventral and dorsal limbs separated by relatively spared central parenchyma (Figure 1). A rostral notch without complete separation of the limbs was also classified as present. Each case was classified as “present” or “absent” by consensus between the two investigators; cases in which consensus could not be reached were adjudicated by a third senior investigator (HZG). Independent ratings were not recorded separately; therefore, interobserver agreement could not be quantified. This binary variable is the primary outcome of this analysis.

### Variable coding and Statistical methods

Because terminology in the source literature was highly heterogeneous, pre-defined coding rules were developed before data extraction. Fistula location was classified as CCJ/C1/foramen magnum, intracranial dural sinus (e.g., superior petrosal sinus, tentorium, or transverse-sigmoid sinus), lower cervical nerve-root sleeve (C5, C6, or C8), or other/unclear. Feeding arteries were categorized according to the predominant arterial supply as vertebral artery meningeal/radicular branch, ascending pharyngeal artery, occipital artery, middle meningeal artery, internal carotid artery/meningohypophyseal trunk, or multiple/mixed feeders (5, 6). Venous drainage direction was defined relative to the CCJ, following the terminology of previous CCJ dAVF series (5, 7). Ascending drainage was defined as arterialized venous flow directed rostrally along the cord surface toward the CCJ and the brainstem venous system, described in the original report as “ascending,” “rostral,” “cranial,” or “upward.” Descending drainage was defined as flow directed caudally along the spinal cord away from the CCJ, either explicitly described as “descending,” “caudal,” “inferior,” or “downward,” or described as extending caudally to the thoracic cord; for intracranial fistulas, this included drainage descending from the posterior fossa through the foramen magnum into the spinal pial veins. Bidirectional drainage was assigned when both rostral and caudal components were explicitly described. “Perimedullary drainage” refers to drainage into the pial venous network on the surface of the medulla and spinal cord, namely the anterior and posterior median veins and the coronal venous plexus (8); because this term describes the drainage territory rather than its direction, cases described only as perimedullary were classified as unspecified. Drainage was coded from the text and angiographic figures of the original reports, by consensus between two investigators. Symptom duration was dichotomized at 1 month into acute and subacute/chronic presentations. Treatment was categorized as surgery, endovascular embolization, combined treatment, or other. Clinical outcome was classified as good/near-complete recovery, partial improvement, poor/no improvement, or unclear according to the terminology used in the original reports. Age was analyzed as a continuous variable; when reported only by decade (e.g., “60s”), the midpoint of the interval was used for analysis. All 64 cases were included in the estimate of the prevalence of the snake-head sign; cases in which a given variable was not reported were excluded from the analysis of that variable only.

Given the rarity of CCJ-region dAVFs and the retrospective literature-based nature of this study, statistical analyses were considered exploratory and hypothesis-generating. Categorical variables were compared using Fisher exact test or chi-square test as appropriate, and continuous variables were analyzed using *t*-test. Effect estimates were interpreted cautiously, particularly when confidence intervals were wide because of limited sample size.

## Results

### Study population and prevalence of the snake-head sign

The literature search identified 64 eligible cases of dAVF involving the CCJ venous system. Among these cases, the snake-head sign was identified in 31 patients (48.4%) based on the available sagittal T2-weighted or FLAIR images. The sign was observed across a range of fistula locations and vascular configurations, suggesting that it represents a relatively common imaging phenotype among reported CCJ-related dAVF cases rather than a feature restricted to a specific anatomical subtype.

Clinical and angiographic characteristics according to the presence or absence of the snake-head sign are summarized in Table 1. No significant differences were observed between patients with and without the sign regarding age, sex, symptom duration, feeding artery, treatment modality, or clinical outcome (Table 1).

**Table 1:** Comparison of clinical and imaging characteristics between patients with and without the snake-head sign.

Characteristic	Sign present (*n* = 31)	Sign absent (*n* = 33)	OR (95% CI)	Pairwise *P*	Overall *P*
Age (years, mean ± SD)	56.8 ± 14.9 (*n* = 29)	59.5 ± 13.1 (*n* = 28)	—	—	0.507^*^
Sex: female	9 (29.0%)	6 (18.2%)	—	0.554	—
Symptom duration ≤ 1 month (acute)	12 (60.0%)	9 (37.5%)	2.50 (0.74–8.45)	0.225	—
Fistula location					0.195
Lower cervical nerve-root sleeve (C5/C6/C8)	4 (100.0%)	0 (0%)	10.96 (0.57–212.6)	0.050	
CCJ/C1/foramen magnum	15 (42.9%)	20 (57.1%)	0.61 (0.23–1.64)	0.451	
Intracranial dural sinus (SPS/tentorial/transverse-sigmoid, etc.)	11 (50.0%)	11 (50.0%)	1.10 (0.39–3.09)	1.000	
Other/unclear	1 (33.3%)	2 (66.7%)	0.52 (0.04–6.00)	1.000	
Draining vein direction					0.062
Ascending/converging on CCJ	4 (100.0%)	0 (0%)	10.64 (0.55–206.4)	0.053	
Descending/away from CCJ	4 (26.7%)	11 (73.3%)	0.28 (0.08–1.02)	0.075	
Bidirectional	1 (50.0%)	1 (50.0%)	1.03 (0.06–17.3)	1.000	
Unspecified (perimedullary only)	22 (52.4%)	20 (47.6%)	1.47 (0.51–4.21)	0.595	
Not reported	0	1	—	—	
Feeding artery					0.684
Vertebral artery (meningeal/radicular branch)	10 (45.5%)	12 (54.5%)	0.83 (0.30–2.34)	0.796	
Ascending pharyngeal artery	3 (75.0%)	1 (25.0%)	3.43 (0.34–34.9)	0.347	
Multiple/mixed feeders	10 (50.0%)	10 (50.0%)	1.10 (0.38–3.15)	1.000	
Other single feeders combined	8 (44.4%)	10 (55.6%)	0.80 (0.27–2.39)	0.784	
Treatment					0.886
Surgery	11 (47.8%)	12 (52.2%)	0.96 (0.35–2.67)	1.000	
Embolization (TAE/TVE)	15 (53.6%)	13 (46.4%)	1.44 (0.53–3.89)	0.615	
Combined or other treatment	5 (38.5%)	8 (61.5%)	0.60 (0.17–2.09)	0.539	
Outcome					0.564
Good/near-complete recovery	10 (58.8%)	7 (41.2%)	1.77 (0.57–5.44)	0.400	
Poor/no improvement	1 (25.0%)	3 (75.0%)	0.33 (0.03–3.39)	0.614	
Partial improvement or unclear	20 (46.5%)	23 (53.5%)	0.79 (0.28–2.25)	0.791	

Percentages are row percentages, that is, the proportion of cases within each category that demonstrated or did not demonstrate the snake-head sign.

Odds ratios and pairwise *P*-values compare the category shown with all remaining cases of the same variable (Fisher exact test); a continuity correction of 0.5 was applied when a zero cell was present. These pairwise comparisons are exploratory and were not corrected for multiple testing.

Overall *P*-values compare all categories of the variable (chi-square test): fistula location and drainage direction, four groups each; feeding artery, seven groups, analyzed with each feeder category separately although the less frequent categories are combined in the table for clarity; treatment and outcome, four groups each.

^*^Two-sample t-test. Drainage direction was not reported in one case, which was excluded from the corresponding analysis; age and sex were not reported in some cases, and denominators therefore differ between variables.

### Association between venous drainage characteristics and the snake-head sign

Among the examined variables, the most notable differences were observed for venous drainage direction. In cases where the draining vein direction was explicitly described, all four cases with cranially directed venous drainage toward the CCJ venous network demonstrated the snake-head sign (100%). In contrast, among 15 cases with a purely caudally directed draining vein extending away from the CCJ toward the thoracic cord, the sign was present in four cases (26.7%). The difference between these groups was not statistically significant (Fisher exact test, OR = 0.28, 95% CI 0.08–1.02). Among 42 cases without explicit description of venous direction, in which the drainage pattern was generally reported as perimedullary, the snake-head sign was present in 22 cases (52.4%; Figure 3A). Bidirectional drainage was described in two cases, one of which demonstrated the sign, and drainage information was not reported in one case.

**Figure 3:**
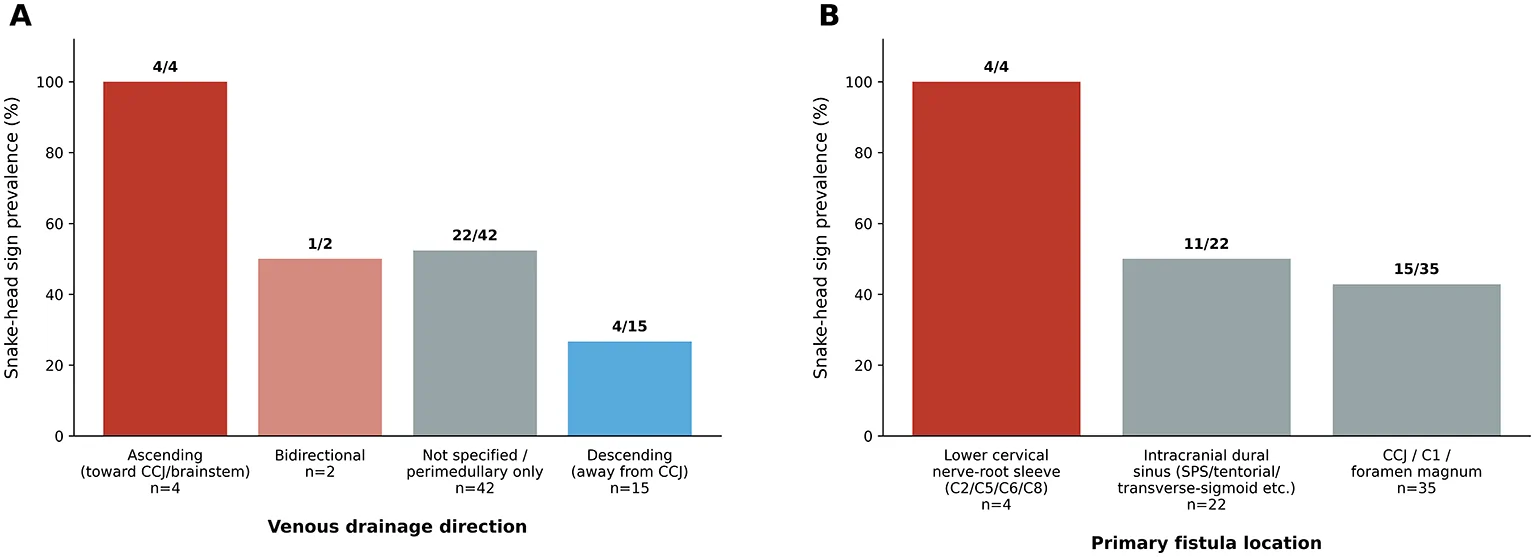
Prevalence of the snake-head sign according to venous drainage direction and fistula location. **(A)** Prevalence by venous drainage direction. **(B)** Prevalence by primary fistula location. Numbers above the bars indicate cases with the snake-head sign/total cases in each category. CCJ, craniocervical junction; SPS, superior petrosal sinus.

### Association between fistula location and the snake-head sign

Fistula location also demonstrated differences in the prevalence of the snake-head sign. All four cases with fistulas arising from lower cervical nerve-root sleeves (C5, C6, or C8) demonstrated the sign (100%). Among cases with fistulas located at the CCJ, C1, or foramen magnum, the sign was present in 15 of 35 cases (42.9%). Among cases involving higher intracranial dural sinuses, including the superior petrosal sinus, tentorial region, and transverse-sigmoid sinus, the sign was observed in 11 of 22 cases (50.0%; Figure 3B).

### Exploratory analysis of factors associated with the snake-head sign

The forest plot summarizes exploratory associations between selected clinical and imaging variables and the presence of the snake-head sign (Figure 4). Most evaluated variables demonstrated wide confidence intervals, reflecting substantial uncertainty due to the limited number of reported cases. Variables related to venous drainage pattern and fistula location showed the strongest differences, whereas demographic characteristics, clinical duration, arterial supply, treatment modality, and outcome measures did not demonstrate clear associations.

**Figure 4:**
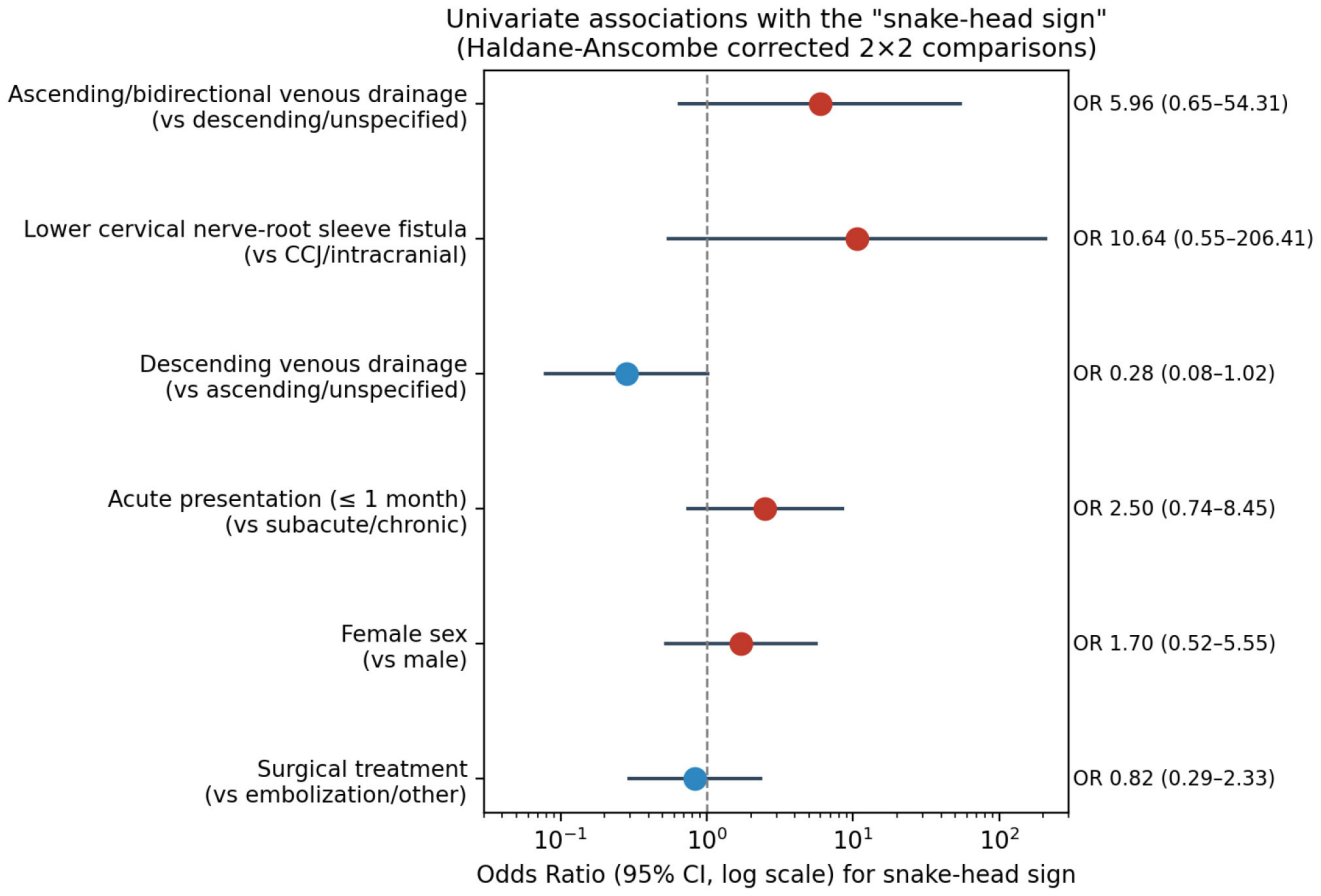
Forest plot of univariate associations between key clinical/imaging variables and the snake-head sign.

## Discussion

The principal finding of this study is the identification of the snake-head sign as a previously uncharacterized MRI feature in patients with CCJ-region dAVFs. Nearly half of the reported cases demonstrated this characteristic pattern of medullary and upper cervical cord edema, suggesting that it is not an isolated imaging curiosity but a relatively common radiographic phenotype. Because CCJ-region dAVFs often present with non-specific symptoms and are frequently mistaken for inflammatory, degenerative, or other non-vascular myelopathies, recognition of this feature may increase diagnostic awareness and prompt timely vascular investigation (1, 9). For neurologists who do not routinely evaluate spinal vascular disorders, the snake-head sign may serve as a simple visual cue that raises suspicion for an underlying dAVF rather than establishing the diagnosis itself.

From a functional anatomical perspective, venous drainage of the medulla and upper cervical cord is organized longitudinally rather than segmentally. Intrinsic medullary and spinal veins drain radially into a pial network on the surface of the cord, namely the anterior and posterior median veins and the coronal venous plexus. This network runs continuously from the spinal cord to the ventral brainstem, where it continues as the anterior median medullary and anterior pontomesencephalic veins (8, 10, 11). The pial network drains outward through radiculomedullary and emissary-bridging veins; at the foramen magnum, the bridging vein of the medulla opens laterally into the condylar vein, and at the CCJ the condylar and emissary veins connect the intracranial sinuses with the vertebral venous plexus (11–13). Dural shunts in the lateral epidural compartment, which includes the spinal nerve-root sleeves, the foramen magnum (marginal sinus), and the petrosal and tentorial regions, drain directly into these bridging veins and therefore always reflux into the perimedullary or cortical veins; shunts in other compartments may acquire the same drainage when their normal sinus outflow is restricted (13). Arterialization then raises pressure in the intrinsic veins that share this outflow, reducing the arteriovenous pressure gradient and producing congestive edema (14). This shared pial outflow may explain why fistulas at anatomically distant sites produce similar congestion at the CCJ, and why the distribution of edema reflects the venous route rather than the shunt location.

The present findings suggest two complementary, non-exclusive mechanisms by which this imaging pattern may arise. Unlike conventional descriptions that focus primarily on the arterial origin of the fistula, the snake-head sign appears to be more closely related to the configuration of venous outflow. The CCJ represents a unique transition zone where intracranial and spinal venous pathways converge within a relatively confined anatomical space: the anterior median medullary vein continues caudally as the anterior spinal vein and communicates with the dural sinuses through bridging veins (7, 11). First, when abnormal venous drainage converges cranially toward the CCJ, changes in flow direction together with local anatomical constraints may promote focal venous remodeling, such as dilatation, tortuosity, or varix formation of the draining veins, or narrowing at their dural exit. Local venous anatomy is thought to shape outflow at this level: the densely trabeculated C1–C2 venous system has been proposed to offer high resistance and to favor upward drainage, and high-flow CCJ fistulas may develop venous ectasia or varices exerting mass effect on the brainstem (7). Varices on the draining veins are common in CCJ-AVFs and were observed in 41% of lesions in a multicenter cohort and pooled analysis (15). Such remodeling could impede outflow and create a focal zone of elevated venous pressure at the CCJ, although this remains unproven; notably, varices were not significantly associated with myelopathy in that series (15). Second, because the sign itself represents intramedullary edema rather than a vascular structure, this focal venous hypertension would be expected to be transmitted to the medulla and upper cervical cord, producing edema concentrated in this segment. The divided rostral configuration, with ventral and dorsal involvement and relative sparing of the central medulla, might correspond to congestion of the ventral and dorsal surface venous networks into which the medulla drains. In this framework, venous remodeling would represent the upstream hemodynamic change and the congestive edema pattern its imaging expression. Previous studies have linked ascending drainage mainly to hemorrhagic presentation (7, 9, 15, 16); because hemorrhagic lesions were excluded from the present analysis, our cases with ascending drainage represent its non-hemorrhagic subset. Both components remain speculative, and the observed distribution of the sign across different venous drainage patterns and fistula locations is compatible with, but does not establish, this interpretation.

Our angiographic data support this interpretation. The draining-vein direction analyzed in Table 1 was extracted from the angiographic descriptions of the original reports. The sign was present in all cases in which arterialized drainage ascended along the cord surface toward the CCJ. In these cases, the venous outflow converges on the ventral medullary venous network, and this narrow transition zone becomes the point of maximal venous hypertension. For example, in a C8 root-sleeve fistula, the ascending intradural vein divided at the CCJ into the median anterior spinal and anterior medullary veins, with congestion of the vein of the cerebellomedullary fissure and edema confined to the medulla (17). By contrast, when drainage descended along the anterior spinal vein toward the thoracic cord, congestion extended caudally along the cord rather than concentrating at the CCJ, and the sign was uncommon. Because spinal and brainstem venous drainage is longitudinal and non-segmental, the sign reflects where venous outflow converges rather than where the shunt is located, and it cannot replace catheter angiography for fistula localization.

An important implication of these findings is that the snake-head sign may represent a venous congestion phenotype rather than a marker of fistula subtype or arterial anatomy. No clear relationships were identified between the sign and patient demographics, symptom duration, feeding artery, treatment strategy, or clinical outcome, suggesting that its occurrence is unlikely to reflect disease severity or a particular angioarchitectural classification (1, 18). Instead, the sign appears to reflect where venous congestion concentrates in response to abnormal shunt flow. This distinction may be clinically relevant because radiographic manifestations of dAVFs are often interpreted according to arterial anatomy, whereas the present findings suggest that venous remodeling and congestion may also generate reproducible imaging patterns that provide complementary diagnostic information.

The clinical role of the snake-head sign should therefore be viewed primarily as a hypothesis-generating imaging observation rather than a validated diagnostic biomarker. When recognized on routine sagittal MRI, particularly in patients with otherwise unexplained venous congestive myelopathy, it may encourage more careful evaluation of abnormal perimedullary vessels and consideration of dedicated vascular imaging, including catheter angiography when clinically appropriate. Importantly, because the sign indicates the distribution of venous congestion rather than the location of the shunt, and was observed with fistulas ranging from intracranial dural sinuses to the lower cervical root sleeves, it cannot be used to localize the fistula, and catheter angiography of all potential feeders remains mandatory. Prospective multicenter studies incorporating standardized MRI assessment, blinded image interpretation, and systematic angiographic or surgical correlation will be required to establish its diagnostic performance, reproducibility, and underlying anatomical basis.

Several limitations should be acknowledged. First, the study was based on a relatively small number of published cases, and some key variables, particularly draining-vein direction, were explicitly described in only a minority of reports. Consequently, several effect estimates were associated with wide confidence intervals and should be interpreted cautiously. Second, the sign was assessed on published images, which often represent single slices selected by the original authors, rather than on original imaging datasets. Moreover, the sign was classified by consensus and independent ratings were not recorded, so interobserver agreement could not be quantified. The reproducibility of the sign, particularly among general radiologists and neurologists, therefore remains to be established in prospective studies with blinded independent readers. Third, angiographic variables were extracted from free-text descriptions in the original literature, and differences in reporting practices may have introduced classification bias. Fourth, the included cases were acquired over different time periods using heterogeneous MRI platforms and imaging protocols, which may have influenced the detectability of subtle vascular findings. Finally, because this retrospective literature-based analysis was designed to generate rather than confirm hypotheses, the proposed mechanism of venous remodeling remains inferential and requires validation in prospective studies incorporating standardized imaging review and direct angiographic correlation.

In conclusion, the snake-head sign is a recognizable MRI feature observed in a substantial proportion of reported CCJ-region dAVF cases. Although its diagnostic performance and reproducibility require prospective validation, recognition of this characteristic pattern of medullary and upper cervical cord edema may provide an additional clue for clinicians evaluating patients with unexplained venous congestive myelopathy (2). Although the sign was more frequent in cases with cranially convergent venous drainage, this difference did not reach statistical significance and was based on a small number of cases. It therefore only raises, rather than supports, the hypothesis that the snake-head sign is related to the venous outflow pattern at the CCJ rather than to a specific arterial source or fistula subtype. Future multicenter studies incorporating standardized MRI assessment and angiographic correlation are needed to determine its diagnostic utility and clarify the underlying venous mechanisms.

## Data Availability

The original contributions presented in the study are included in the article/Supplementary material, further inquiries can be directed to the corresponding author.
